# Discovery of non-invasive biomarkers for the diagnosis of endometriosis

**DOI:** 10.1186/s12014-019-9235-3

**Published:** 2019-04-06

**Authors:** Stella Irungu, Dimitrios Mavrelos, Jenny Worthington, Oleg Blyuss, Ertan Saridogan, John F. Timms

**Affiliations:** 1 Department of Women’s Cancer, Institute for Women’s Health, University College London, Cruciform Building 1.1, Gower Street, London, WC1E 6BT UK; 2 Reproductive Medicine Unit, University College London Hospital, Elizabeth Garrett Anderson Wing, Lower Ground Floor, 235 Euston Road, London, NW1 2BU UK

**Keywords:** Endometriosis, Biomarkers, Eutopic tissue, Ectopic tissue, Serum

## Abstract

**Background:**

Endometriosis is a common gynaecological disorder affecting 5–10% of women of reproductive age who often experience chronic pelvic pain and infertility. Definitive diagnosis is through laparoscopy, exposing patients to potentially serious complications, and is often delayed. Non-invasive biomarkers are urgently required to accelerate diagnosis and for triaging potential patients for surgery.

**Methods:**

This retrospective case control biomarker discovery and validation study used quantitative 2D-difference gel electrophoresis and tandem mass tagging–liquid chromatography–tandem mass spectrometry for protein expression profiling of eutopic and ectopic endometrial tissue samples collected from 28 cases of endometriosis and 18 control patients undergoing surgery for investigation of chronic pelvic pain without endometriosis or prophylactic surgery. Samples were further sub-grouped by menstrual cycle phase. Selected differentially expressed candidate markers (LUM, CPM, TNC, TPM2 and PAEP) were verified by ELISA in a set of 87 serum samples collected from the same and additional women. Previously reported biomarkers (CA125, sICAM1, FST, VEGF, MCP1, MIF and IL1R2) were also validated and diagnostic performance of markers and combinations established.

**Results:**

Cycle phase and endometriosis-associated proteomic changes were identified in eutopic tissue from over 1400 identified gene products, yielding potential biomarker candidates. Bioinformatics analysis revealed enrichment of adhesion/extracellular matrix proteins and progesterone signalling. The best single marker for discriminating endometriosis from controls remained CA125 (AUC = 0.63), with the best cross-validated multimarker models improving the AUC to 0.71–0.81, depending upon menstrual cycle phase and control group.

**Conclusions:**

We have identified menstrual cycle- and endometriosis-associated protein changes linked to various cellular processes that are potential biomarkers and that provide insight into the biology of endometriosis. Our data indicate that the markers tested, whilst not useful alone, have improved diagnostic accuracy when used in combination and demonstrate menstrual cycle specificity. Tissue heterogeneity and blood contamination is likely to have hindered biomarker discovery, whilst a small sample size precludes accurate determination of performance by cycle phase. Independent validation of these biomarker panels in a larger cohort is however warranted, and if successful, they may have clinical utility in triaging patients for surgery.

**Electronic supplementary material:**

The online version of this article (10.1186/s12014-019-9235-3) contains supplementary material, which is available to authorized users.

## Introduction

Endometriosis is estimated to affect 5–10% of women of reproductive age. Although some women with endometriosis may be asymptomatic, it can cause significant pain symptoms and infertility. The diagnosis of endometriosis can be difficult to achieve. Various studies worldwide have documented a delay of 7–11 years from appearance of symptoms until diagnosis [1–3]. This delay is partly due to the fact that an invasive laparoscopy is needed to establish the diagnosis in some women. Imaging techniques are sensitive for diagnosis of ovarian endometriomas and deep endometriosis in experienced hands, but are less accurate for diagnosing other forms of endometriosis [4].

While efforts have been made to develop blood-based diagnostic tests for endometriosis, few reported markers have been independently validated and none are in clinical use. As a result, definitive diagnosis of endometriosis remains surgical, requiring laparoscopy under general anaesthetic, exposing patients to potentially serious complications. Difficulty in establishing a diagnosis leads to patients spending years on visits to their doctors and unnecessary investigations, adding to the significant economic burden of the disease [5]. While early stage endometriosis is not without consequence, delay in diagnosis may allow disease progression with reduced treatment efficiency and poorer outcomes. There is clearly an urgent and unmet need for a minimally invasive diagnostic test for endometriosis, though clinically useful blood-borne markers have yet to be found [6, 7]. The aim of a non-invasive test of endometriosis should be to expedite clinical diagnosis by achieving a high positive predictive value (> 90%) or to triage patients for laparoscopic investigation for conclusive pathological diagnosis. A lower sensitivity may be tolerated for the latter if specificity is high (> 90%). Ideally, a test would also need to function independently of menstrual cycle phase. Early diagnosis of endometriosis may allow preventative measures that delay progression of the disease such as use of the progesterone only pill or Mirena coil. Women hoping to conceive or those who are refractory to medical management can be put forward for surgical treatment earlier.

The first aim of our study was to identify potential biomarkers through proteomic profiling of eutopic and ectopic endometrial tissue specimens taken from women with a confirmed diagnosis of endometriosis and relevant controls undergoing exploratory surgery for chronic pelvic pain without endometriosis or prophylactic surgery due to strong familial risk of cancer. The second aim was to test these potential biomarkers in sera from a larger case control set of women, and additionally validate biomarkers reported in the literature [4].

## Materials and methods

### Patient recruitment

Patients and samples were sourced from the University College London Hospital Gynaecology Department following ethical approval and informed consent. Patients were those referred by their general practitioners or other clinicians for investigation of pelvic pain or for diagnosis and/or treatment of endometriosis. Those who agreed to undergo laparoscopic surgery for investigation and treatment of endometriosis, pelvic pain or bilateral salpingoophorectomy for strong family history of breast and ovarian cancer were approached and recruited. Inclusion criteria were as follows: ‘endometriosis cases’ were defined as women diagnosed with endometriosis at laparoscopy and confirmed histologically; ‘controls with pain’ were defined as symptomatic women with pelvic pain of unknown cause or chronic pelvic inflammatory disease without surgical evidence of endometriosis; ‘controls without pain’ were regularly cycling women with no known disease undergoing bilateral tubal ligation and/or prophylactic bilateral salpingoophorectomy due to familial risk of breast and ovarian cancer and with no visual evidence of endometriosis at laparoscopy. The following women were excluded from the study; post-menopausal women, women with a positive pregnancy test or unknown pregnancy status on day of surgery, those with other benign conditions or malignancies (particularly patients with fibroids and/or cancer were excluded as these conditions may compromise the integrity of the endometrium), women on any hormonal medication < 3 months prior to surgery and those whose surgical findings and pathological reports were inconsistent. Cycle phase was determined by a triple approach to ensure accuracy; chronologically, by histological dating and by sex steroid hormone determination. Women with unconfirmed menstrual cycle stage were excluded. Additional patient data was collected including age, fertility history, treatment history (oral contraceptive and GnRH analogue use), menstrual cycle phase, pain history, histopathology findings and anatomic characteristics of disease lesions. All patient records were handled per NHS confidentiality practices. Samples were anonymised and sequentially numbered. A lab coding system and database were developed for recording anonymised patient and sample information.

### Sample collection

Endometrial tissue biopsies (eutopic endometrium) were obtained by Pipelle cannula or curettage from women undergoing laparoscopy. Ectopic endometrial tissue (endometriosis) samples were obtained from the same women at laparoscopy. Superficial lesions and deep infiltrating endometriosis samples only were used in this study, the rationale being that imaging techniques are sensitive enough for diagnosis of ovarian endometriomas, but are less accurate for diagnosing endometriosis elsewhere in the pelvis.

A portion of each tissue specimen was fixed in 10% buffered formalin for histological examination, whilst remaining tissue was washed in sterile phosphate buffered saline to remove excess blood. Tissues were dried using lint-free paper, transferred into weighed Eppendorf tubes and snap-frozen in liquid nitrogen. Samples were transported to the lab and stored at − 80 °C. Cycle stage for each patient was determined by endometrial dating by an experienced histopathologist without prior knowledge of sample group according to [8]. Endometriotic lesions were confirmed histologically. Samples were excluded where there was insufficient tissue (< 20 mg wet weight). 10 mL of blood was also collected from each patient before surgery by venepuncture into two vacutainer gel tubes. 5 mL of blood was used for determination of oestradiol, progesterone, CA125 and CRP levels using standard assays. The remaining sample was allowed to clot at room temperature for 1 h, centrifuged at 3000*g* for 10 min at 4 °C, the serum collected, aliquoted and stored at − 80 °C. In total, 87 women met the inclusion criteria who had donated a serum sample; 45 with endometriosis, 21 controls with pain and no evidence of endometriosis and 21 controls with no pain and no disease (Table 1).

**Table 1:** Characteristics of subjects included in the study and clinical grouping

	Control	Pain	Endometriosis
Number	21	21	45
Age (years)			
Mean (SD)	37.5 (8.5)	32.2 (6.5)	35.9 (5.8)
Median (range)	36 (20–51)	33 (19–48)	36.0 (20–52)
Symptoms			
Subfertility	4	8	16
Pain	0	22	38
Cycle phase			
Proliferative (1)	10	9	13
Secretory (2)	9	12	26
Inactive (0)	2	0	6

### Protein extraction, quality assessment, immunodepletion and pooling

Each snap-frozen tissue sample was weighed and homogenised by grinding in liquid nitrogen into a fine powder. Ground tissue was lysed in 2D lysis buffer (8 M urea, 4% w/v CHAPS and 10 mM Tris–HCl pH 8.3) using a ratio of 5 μL of lysis buffer per 1 mg of tissue at room temperature to extract protein. Samples were sonicated and centrifuged at 3000*g* for 10 min at 4 °C to pellet insoluble material. The supernatant was collected and protein concentration determined using a Bradford microtitre plate assay (Pierce) using a bovine serum albumin standard curve. All samples were normalised to the same (lowest) protein concentration using 2D lysis buffer. To assess quality of the samples, 10 μg total protein from each sample were run on NuPAGE^®^ Novex^®^ 4–12% Bis–Tris 1.5 mm, pre-cast SDS-PAGE gels (Invitrogen) alongside 10 μg of total protein from human serum. Gels were stained with colloidal Coomassie Blue (Instant Blue gel stain; Expedion) and imaged on a GS-800 densitometer (BioRad). Samples were excluded if they comprised largely of serum proteins (i.e. barely visible tissue protein bands) and/or had low protein staining above 20 kDa. Equal protein amounts from each sample were then pooled into 6 groups according to tissue type, case control status and menstrual cycle phase. These groups were: eutopic tissue from endometriosis cases in the secretory (ES; n = 19) or proliferative phase (EP; n = 9), eutopic tissue from patients with chronic pelvic pain with no evidence of endometriosis at laparoscopy in the secretory phase (PS; n = 7), eutopic tissue from asymptomatic controls scheduled for risk-reducing surgery with no evidence of disease at laparoscopy in the secretory (CS; n = 6) or proliferative phase (CP; n = 7) and ectopic tissue from endometriosis cases in the secretory phase (EcS; n = 11). There were no eutopic samples available for the pain group in proliferative phase or eligible ectopic samples from endometriosis patients in proliferative phase. To improve proteomic coverage, immunodepletion of the 12 most abundant serum proteins was carried out on the pooled samples using Protein Purify 12 (PP12) Human Serum Protein Immunodepletion resin (R&D). Briefly, 500 μL of PP12 immunodepletion slurry was incubated with diluted endometrial tissue lysate (500 μg protein) on a rotary shaker for 30 min. Unbound material was recovered using Spin-X Filter units by centrifugation, the filtrates were concentrated to 25 μL using 5 kDa molecular weight cut-off Vivaspin columns and protein concentration determined using the Bradford method.

### 2D-DIGE profiling of endometrial tissue lysates

Immunodepleted samples (80 μg total protein) were labelled differentially in triplicate with NHS-cyanine dyes (GE Healthcare) at a dye to protein ratio of 6 pmol/μg protein on ice for 30 min in the dark. Cy2 dye was used to label an internal standard pool prepared by mixing equal amounts of protein from all pools. Reactions were quenched by adding a 20-fold molar excess of l-lysine to dye and incubating on ice for 10 min in the dark. Pairs of differentially labelled samples (Cy3 and Cy5) and Cy2-labelled standard were mixed appropriately and reduced by addition of dithiothreitol (DTT) (65 mM final concentration) and carrier ampholines and pharmalytes added to a final concentration of 2% v/v with 1 μL of 2% bromophenol blue. Samples were then run on 9 2D-gels according to our standard procedures [9]. Briefly, Immobiline IPG strips (24 cm; pH 3–10 NL) (GE Healthcare) were rehydrated with labelled samples overnight and isoelectric focusing performed for 80 kVh at 16 °C. Strips were equilibrated with DTT reduction and iodoacetamide (IAM) alkylation steps, transferred onto 10% SDS-PAGE bonded gels cast between low fluorescence glass plates, overlaid with agarose and run for 16 h at 2.2 W per gel. Images were acquired by scanning gels on a Typhoon 9400 multi-wavelength fluorescence imager (GE Healthcare) and analysed using DeCyder Software V6 (GE Healthcare), calculating standardised spot abundances (using the Cy2-labelled standard pool) for all matched spots across the 9 gel images. Differences in spot abundances between conditions were filtered by specifying a 1.5 threshold of average fold-change with *P *< 0.05 (Student’s *t* test). Pick lists of spots of interest were created and exported to an Ettan spot picking robot (GE Healthcare) for excision from the same colloidal Coomassie Blue post-stained gels.

### Protein identification by LC–MS/MS

In-gel digestion was carried out by washing gel pieces three times in 200 µL of 50% acetonitrile (ACN) and then in 200 µL of 100% ACN. Gel pieces were dried in a SpeedVac for 20 min, reduced for 45 min with 20 µL of 10 mM DTT in 5 mM ammonium bicarbonate (AmBiC) pH 8.0 at 50 °C, alkylated with 20 µL of 50 mM IAM in 5 mM AmBiC, washed twice in 200 µL of 50% ACN and dried in a SpeedVac. Gel pieces were then overlaid with 50 ng modified trypsin (Promega) in 10 µL of 5 mM AmBiC and incubated for 16 h at 37 °C. The supernatant was collected and peptides extracted twice with 20 µL of 5% trifluoroacetic acid in 50% ACN and the supernatants pooled. Peptide extracts were vacuum dried, desalted using reversed phase C18 ZipTips™ (Merck Millipore) as per the manufacturer’s instructions, dried down in a SpeedVac and stored at − 20 °C prior to MS analysis. Digested samples were re-suspended in 6 μL of 0.1% (v/v) formic acid (FA) and analysed by LC–MS/MS using an Ultimate 3000 nano-flow reversed phase-liquid chromatography (RPLC) system linked to an LTQ-Orbitrap XL mass spectrometer (Thermo-Scientific) equipped with a Picoview PV550 nano-electrospray ion source (New Objective Inc.). Samples were first injected onto an Acclaim PepMap 100 C18 pre-column (5 μm, 100 Å, 300 μm i.d × 5 mm) (Thermo Fisher) and washed for 3 min with 90% buffer A (H_2_O + 0.1% (v/v) FA) at a flow rate of 25 μL/min. Chromatographic separation was then performed on an Acclaim PepMap 100 C18 nano-LC column (3 μm, 100 Å, 75 μm i.d × 25 cm) (Thermo Fisher) with a 60 min linear gradient of 10–50% buffer B (100% ACN + 0.1% (v/v) FA) at a flow rate of 300 nL/min. Tandem MS was performed in the data-dependent mode to automatically switch between MS (full ion scan) and MS/MS (fragment ion scan) acquisition. Survey full scan MS spectra (*m/z* 390–1700) were acquired in the orbitrap with a resolution of 60,000 at *m/z* 400. The most intense (top 6 ions per survey scan) were sequentially isolated for fragmentation in the linear ion-trap by collision induced dissociation and dynamically excluded for 60 s. Acquired mass spectra were processed using Mascot Distiller version 2.5 (Matrix Science Ltd) and searched against the SwissProt database. The following parameters and search filters were used; MS tolerance was 10 ppm, MS/MS tolerance was 0.5 Da, two missed cleavages were allowed, carbamidomethylation of cysteines was set as a fixed modification, methionine oxidation, acetylation (protein N-term) and deamidation (asparagine and glutamine) were set as variable modifications. Protein identifications were accepted where there were two unique peptides of score > 20 at a Mascot significant threshold of *P *< 0.05. Protein identifications were matched to specific spots in DeCyder with experimental molecular weights checked against theoretical values.

### Profiling of endometrial tissues using a TMT-based 3D–LC–MS/MS strategy

A 6-plex tandem mass tagging (TMT) approach with 3-dimensional peptide separation [strong anion exchange (SAX) chromatography, off-line high pH RPLC and low pH nano-RPLC coupled to MS/MS] was applied to the 6 tissue lysate pools described above to maximise quantitative proteomic coverage. Immunodepleted tissue lysate pools (100 μg each) were re-suspended in 100 mM triethylammonium bicarbonate (TEAB), pH 8.5 and 0.1% SDS, reduced with 1 mM tris(2-carboxyethyl)phosphine for 1 h at 55 °C and alkylated with 7.5 mM IAM for 1 h at RT in the dark. Samples were digested overnight at 37 °C using 4 µg of modified porcine trypsin. Samples were then labelled with TMT reagents (Thermo Fisher). Briefly, each digested pool was labelled with 0.8 mg of one of six TMT reagents (re-suspended in 41 μL of ACN) for 1 h at RT as follows: CS—TMT126, CP—TMT127, PS—TMT128, EcS—TMT129, ES—TMT130 and EP—TMT131. Samples were then incubated with 0.25% hydroxylamine for 30 min at RT to quench the reaction. The six TMT-labelled samples were then combined. SDS was removed using detergent removal spin columns (Pierce) as per the manufacturer’s instructions, the samples desalted using 1 cc Oasis HLB cartridges (Waters) as per the manufacturer’s instructions, dried in a SpeedVac and re-suspended in 300 μL of 100 mM TEAB pH 8.5.

For SAX chromatography, DEAE ceramic HyperD F slurry (300 μL; Pall Corporation) was added to a spin filter unit and centrifuged at 3000 rpm for 2 min to remove storage buffer, then washed with 200 μL of 1 M NaCl in 100 mM TEAB pH 8.5, then three times with 200 µL of 200 mM TEAB pH 8.5 and then equilibrated by washing with 100 mM TEAB pH 8.5, followed by centrifugation at 3000 rpm for 2 min to remove the supernatant. The pooled sample was then incubated with the resin on a rotary shaker for 5 min. Un-bound peptides were removed by centrifugation followed by washing with 200 µL of 100 mM TEAB and the two fractions combined as the flow-through. Bound peptides were then sequentially eluted using 200 μL × 2 of increasing salt concentration buffer; 100 mM TEAB plus 400 mM NaCl, 600 mM NaCl and 1 M NaCl. The two eluates at each salt concentration were combined and desalted using 1 cc Oasis HLB cartridges, vacuum dried and stored at − 20 °C. For high pH RP-LC, peptide fractions were re-suspended in 45 μL of 20 mM ammonium formate pH 8.5 and fractionated on a Poroshell 300SB-C18 (5 μm, 2.1 × 75 mm) column using an Agilent 1100 series microflow pump. Briefly, 40 µL of re-suspended peptides were injected onto the column in 20 mM ammonium formate pH 8.5 and peptides fractionated using a 0–45% linear gradient of 20 mM ammonium formate pH 8.5 in 80% acetonitrile at a flow rate of 200 µL/min for 55 min. 30 fractions were collected for each of the 4 SAX fractions (total 120 fractions). Each fraction was dried, re-suspended in 200 μL of 0.1% FA and re-dried prior to MS analysis.

LC–MS/MS was carried out essentially as described above, using a 90 min linear gradient of 10–50% buffer B, with data-dependent acquisition of the top 3 ions for fragmentation by both CID and HCD (collision energy 40%) for optimal reporter ion measurement. For HCD, product ions were detected in the orbitrap at a resolution of 7500. Proteome Discoverer version 2.4 was used for protein identification and quantification using Mascot for searching the SwissProt database. Search parameters were as described above, except that only one missed cleavage was allowed. A co-isolation threshold of 25% was set in the quantification method to limit the recording of reporter ion ratios from multiple peptides. Protein groups with a ratio above 1.5 or below 0.67 for each comparison were considered to be differentially expressed.

### Gene ontology and pathway enrichment analysis

Differentially expressed proteins were imported into WebGestalt [10] and each clinical group analysed separately for enrichment of GO biological process, molecular function and cellular component, GO Slim terms, protein interaction networks, KEGG pathways and disease association. Significantly enriched terms were identified using a hypergeometric test with a Benjamini–Hochberg (BH) correction at a significance of *P *< 0.05. The top 10 GO terms with the most significant *P* values were reported. In each comparison, the protein lists were analysed separately as up- or down-regulated proteins.

### Selection and verification of candidate biomarkers

For selection of proteins for verification, a biomarker scoring system was devised based on fold-change, TMT reporter ion ratio count, variability, number of unique peptide sequences, whether they were possible serum contaminants and their membership in 1 of 5 expression clusters. Clustering was performed using Graphical Proteomics Data Explorer (GProX) based on reporter ion ratios for the different comparisons with a higher score given to candidates whose expressions differed between endometriosis and both control groups and in both cycle phases. Selection was also weighted based on prior knowledge of function, proteins known to be secreted and those for which commercial detection reagents were available. LUM, TPM2, CPM, PAEP and TNC were selected from the discovery profiling work.

Promising candidate markers reported in the literature (sICAM1, MCP1, MIF, IL1R2, VEGF and FST) were also tested using commercial ELISA kits. Assays were tested for reproducibility and technical sensitivity to ensure the protein of interest could be accurately detected in serum samples. Optimal dilutions and intra-assay CVs for the assays are shown in Additional file 1: Table S1. Assays were carried out according to the manufacturers’ instructions using the complete study set.

### Statistical analysis

Data analysis was carried using Graphpad Prism software version 5.0.1 and the R statistical software environment. For samples where the protein of interest was determined to be below the limit of detection of the assay, the value of the lowest standard for that assay was used. The Shapiro–Wilk test was applied to test data distribution with an unpaired t test used to compare groups for normally distributed data and the Mann–Whitney test used for non-normally distributed data. For each clinical group, the data was analysed independently of cycle phase and then by cycle phase to assess menstrual cycle dependency. A *P* value of < 0.05 was considered significant. To determine the diagnostic performance of each biomarker, ROC curve analysis was performed with the area under the curve (AUC) reported for each comparison [endometriosis (E) vs. no-pain control (C) and endometriosis vs. pain control (P)]. Multi-variate analysis was carried out to assess the diagnostic performance of combined marker panels using logistic regression, reporting cross-validated (leave-one-out) AUC and sensitivity at fixed specificity.

## Results

### Discovery proteomic profiling by 2D-DIGE

In total, 122 pre-menopausal women were consented to the study with 87 women meeting the inclusion criteria. The set was divided into cases who were diagnosed with endometriosis (n = 45) and two control groups comprising women with pelvic pain (n = 21) or no known disease and no pain (n = 21). Eutopic and ectopic endometrial tissue specimens from these women were lysed and first quality-assessed by SDS-PAGE with colloidal Coomassie Blue staining. It was evident that some samples were heavily contaminated with blood proteins, impairing the ability to visualise tissue-derived proteins at that protein load, or had low protein staining overall (see Additional file 1: Figure S1). These tissue samples were subsequently excluded from further analysis. The remaining samples were pooled (based on equal protein amount) into six groups by clinical group and cycle phase with 6–20 tissue samples pooled per group (designated as CS, CP, PS, ES and EcS—see “Materials and methods” section). With blood protein contamination in mind, immunodepletion of the 12 most abundant serum proteins was undertaken to improve tissue protein coverage (Additional file 1: Figure S1). Contamination was significant, as immunodepletion resulted in protein yields of 20–25% of starting material, although the approach did reveal lower abundance (tissue-derived) proteins.

2D-DIGE profiling was undertaken, analysing the 6 pools in triplicate for differential protein expression. Merged fluorescent gel images are shown in Additional file 1: Figure S2. Differential expression was assessed using Decyder image analysis software, where spot matching was performed and standardised spot abundances calculated with reference to a Cy2-labelled standard pool (equal mix of all samples) run on all gels. Spot abundances were compared between clinical groups revealing 72 protein spots matching on all 9 gel images that displayed a > 1.5-fold change in standardised abundance (*P *< 0.05) between one or more clinical groups. Of these, 52 were detectable as well-defined colloidal Coomassie Blue-stained spots. These spots were excised, digested with trypsin and analysed by LC–MS/MS, resulting in 130 confident protein identifications (see Additional file 1: Table S2).

Since multiple proteins were identified in many of the spots, it was not possible to assign which protein was differentially expressed with absolute certainty. Therefore, the most likely protein was assigned based on the number of matched peptides and concordance of theoretical and experimental molecular weights. Differentially expressed proteins comprised cytoskeletal proteins, metabolic enzymes, extracellular matrix proteins, muscle proteins, those involved in protein folding and blood proteins, including haemoglobin. A simple biomarker score was used to prioritise candidates for further testing and was based on proteins displaying the same directionality of differential expression between endometriosis and both control groups and in ectopic versus eutopic tissue and that did not differ between control groups (PS/CS). High-scoring proteins included lumican (LUM) and tropomyosin β chain (TPM2). Expression of LUM (identified in 3 spots) was higher in the secretory phase endometriosis group compared to both control groups (e.g. spot 708; ES/CS = 1.86, *P *= 0.005; ES/PS = 2.25, *P *= 0.004) and in ectopic compared to eutopic tissue (EcS/ES = 2.09, *P *= 0.008), but not in proliferative phase samples (Additional file 1: Table S2). Similarly, expression of two proteoforms of TPM2 was higher in ectopic versus eutopic tissue and in secretory phase endometriosis compared to control groups [e.g. spot 1548 (EcS/ES = 4.15, *P *= 0.0002; ES/CS = 3.15, *P *< 0.0001; ES/PS = 2.36, *P *= 0.0057)]. These differences suggest menstrual cycle dependency. LUM and TPM2 were selected for further testing as serum biomarkers.

### Discovery proteomic profiling by 3D–LC–MS/MS with TMT labelling

To improve depth of coverage for candidate biomarker discovery, immunodepleted tissue pools were subjected to tryptic digestion, 6-plex TMT labelling and extensive peptide fractionation (120 fractions) using SAX, high pH RPLC and low pH nano RPLC linked on-line to tandem MS. Results are presented in Additional file 2. A total of 1581 proteins groups were identified of which 1433 (91%) had quantitative information across all clinical groups. To gain insight into the possible functional consequences of altered protein expression, a functional enrichment analysis was undertaken using GO biological process and KEGG pathway terms for the differentially expressed proteins (> 1.5-fold). The results were somewhat ambiguous. Analysis of proteins up-regulated in eutopic tissue from endometriosis patients, revealed enrichment of macromolecular complex subunit organization, mRNA metabolic process, protein complex disassembly, translational termination, aromatic/cyclic compound metabolic process, nitrogen compound metabolic process, catabolic process and acute inflammatory response, with only enrichment of mRNA metabolic process shared for all three comparisons (Table 2). Down-regulated proteins were also enriched for macromolecular complex subunit organization, mRNA metabolic process, protein complex disassembly and acute inflammatory response, with no enrichment common to the three comparisons. Comparing ectopic and eutopic tissue, nitrogen compound metabolic process, response to wound healing and cytoskeleton organisation were enriched for up-regulated proteins, whilst mRNA metabolic process and catabolic process were enriched for down-regulated proteins. KEGG pathway mapping revealed enrichment of genes involved in metabolic pathways, ribosome, proteasome, spliceosome and notably, regulation of the actin cytoskeleton, focal adhesions and extracellular matrix (ECM)-receptor interactions, although there was no obvious pattern to the enrichment across the comparisons (Table 2). Disease association enrichment was also ambiguous, although there was a trend of down-regulated gene products linked with carcinoma and neoplasm invasiveness. This is perhaps not surprising since endometriosis shares some features with cancer, such as local and distant invasion, attachment and damage to affected tissues. Numerous muscle-related proteins were identified that were highly expressed in ectopic versus eutopic tissue, including TPM1, 2, 3 and 4, MYLK, MYL6 and 9, PDLIM7, CNN1, CALD1 and TAGLN, suggestive of significant amounts of muscle tissue in the ectopic samples (Additional file 2 and Table 3). ECM proteins including FN1, LUM, COL1A2, COL6A1, COL6A3, COL14A1, PRELP, OGN, DCN, BGN, FMOD and MFAP4, were also highly expressed in ectopic tissue.

**Table 2:** GO enrichment analysis for differentially regulated (≥ 1.5 fold-change) proteins

	ES versus CS		EP versus CP		ES versus PS		EcS versus ES	
	Upregulated	Downregulated	Upregulated	Downregulated	Upregulated	Downregulated	Upregulated	Downregulated
Biological process								
Cellular macromolecular complex subunit organization	5.34E−13	3.16E−05						
mRNA metabolic process	5.34E−13	2.20E−05	0.0099		4.85E−06			1.10E−25
Cellular protein complex disassembly	3.46E−10	3.34E−05						
Protein complex disassembly	4.90E−10							
Translational termination	6.69E−09		0.0044					
Cellular aromatic/cyclic compound metabolic process			0.0092					
Cellular nitrogen compound metabolic process			0.0139				1.92E−10	
Nucleic acid metabolic process					4.85E−06			
Cellular catabolic process					3.42E−05			5.02E−10
Response to wounding							8.51E−14	
Actin filament-based process/cytoskeleton organisation							8.51E−14	
Acute inflammatory response			0.0197	2.58E−06	2.63E−09		1.57E−12	
KEGG pathways								
Metabolic pathways	7.26E−11	4.41E−07				1.40E−05		2.75E−20
Ribosome	4.72E−15							3.29E−16
Focal adhesion	2.21E−05				3.74E−05		1.48E−18	6.72E−32
Regulation of actin cytoskeleton		3.77E−06	4.82E−07				2.48E−11	
ECM-receptor interaction					0.0001	1.40E−05	2.92E−09	
Spliceosome	9.27E−06	9.31E−09			0.0005			
Proteasome	3.55E−05	4.29E−05			1.36E−10			
Oxidative phosphorylation	5.93E−05							4.13E−07
Disease association								
Carcinoma		9.60E−07		2.33E−07		8.97E−05		
Neoplasm invasiveness		7.87E−06					3.61E−08	5.76E−10
Cancer or viral infections		6.40E−06				0.0007		
Anemia, hemolytic	2.15E−13				3.69E−12		1.50E−09	
Adhesion		5.10E−08				1.38E−06	1.39E−18	
Hematologic diseases	1.30E−10							
Protein deficiency	5.19E−08				2.13E−11			2.18E−10
Cardiovascular diseases					5.04E−11		5.54E−10	
Huntington’s disease		1.10E−06			3.87E−15			2.11E−12

Enrichment analysis was performed using WebGestalt. Each clinical group was analysed separately for enrichment of biological process, KEGG pathways and disease association. Significantly enriched terms were identified using a hypergeometric test with a Benjamini-Hochberg (BH) correction with corrected *P* values shown for each comparison

**Table 3:** High-scoring proteins of interest identified by TMT 3D–LC–MS/MS profiling

Acc. No.	Description	Biomarker score	Protein score	Unique peptides	PSMs	Ratio ES/CS	Ratio ES/PS	Ratio EP/CP	Ratio EcS/ES	Ratio PS/CS
P14384	*Carboxypeptidase M CPM*	26	154	2	6	1.619	2.525	2.451	0.319	0.633
P26599	Polypyrimidine tract-binding protein 1 PTBP1	24.6	264	7	11	2.646	2.771	0.953	0.377	0.787
Q14764	Major vault protein MVP	23	151	3	7	0.143	0.158	0.425	10.978	0.894
P29373	Cellular retinoic acid-binding protein 2 CRABP2	23	83	2	3	2.078	1.612	2.544	1.436	1.382
Q01995	*Transgelin TAGLN*	22.2	2119	17	95	1.105	1.324	2.171	18.832	1.240
Q01105	Protein SET	22.2	937	6	29	0.914	0.919	1.084	0.330	0.881
P00915	Carbonic anhydrase 1 CA1	22	5011	14	263	2.086	2.902	0.933	1.285	0.627
O94788	Retinal dehydrogenase 2 ALDH1A2	21.4	301	9	12	0.838	0.898	1.542	0.456	0.903
P16949	Stathmin STMN1	21.2	293	7	16	1.044	0.926	1.411	0.471	0.994
Q13308	Tyrosine-protein kinase 7 PTK7	21.2	362	3	13	0.510	0.677	1.497	0.293	0.807
P06703	Protein S100A6	21	762	5	47	1.379	1.653	0.795	3.364	0.774
P09466	*Glycodelin PAEP*	20.6	698	2	18	0.622	0.863	0.950	0.210	0.705
P02751	*Fibronectin FN1*	20.4	251	8	9	2.706	1.288	0.630	2.104	2.005
Q96KP4	Cytosolic non-specific dipeptidase CNDP2	20.2	1519	16	43	0.724	1.078	0.535	0.333	0.710
P00167	Cytochrome b5 CYB5A	20	692	7	29	0.835	0.880	0.205	0.979	0.948
P06401	*Progesterone receptor PGR*	20	37	1	1	1.415	1.762	2.420	0.321	0.792
P59665	Neutrophil defensin 1 DEFA1	19.6	137	3	11	0.325	2.476	1.315	4.989	0.133
P17661	Desmin DES	19.6	4279	24	216	0.904	0.930	1.381	3.056	1.063
Q05682	Caldesmon CALD1	19.4	677	6	25	1.162	0.950	1.260	7.685	1.223
Q7KZ85	Transcription elongation factor SPT6 SUPT6H	19.4	53	1	6	2.143	3.101	0.960	0.843	0.682
Q9HC84	Mucin-5B MUC5B	19.2	471	12	18	0.193	3.853	0.386	0.650	0.088
P12111	*Collagen alpha*-*3(VI) chain COL6A3*	19	687	12	27	1.933	1.254	1.209	18.478	1.597
P51884	*Lumican LUM*	19	1340	13	51	1.021	0.952	1.216	14.892	1.033
P60660	Myosin light polypeptide 6 MYL6	19	2726	12	92	1.378	0.878	1.148	5.003	1.382
P20774	Mimecan OGN	18.6	731	9	28	0.831	0.798	1.212	20.121	0.905
P09493	*Tropomyosin alpha*-*1 chain TPM1*	18.6	4361	8	186	1.004	1.007	1.245	10.119	0.964
P07951	*Tropomyosin beta chain TPM2*	18.6	4277	6	178	0.988	1.032	1.239	9.162	0.913
P06702	Protein S100A9	18.6	286	3	7	0.406	7.751	2.632	7.255	0.061
P67936	Tropomyosin alpha-4 chain TPM4	18.4	4288	12	201	1.014	1.023	1.230	7.301	0.918
P24821	*Tenascin TNC*	18.4	83	5	5	1.817	1.682	1.502	1.151	1.022
O00264	*PGRMC1*	18.4	191	4	8	1.013	1.035	1.561	0.609	1.228
P52907	F-actin-capping protein subunit a1 CAPZA1	18.2	145	4	7	3.031	1.078	0.885	1.459	1.280
P51888	Prolargin PRELP	18	84	3	5	0.504	0.471	1.466	33.466	1.145
Q05707	*Collagen alpha*-*1(XIV) chain COL14A1*	18	1210	18	49	1.294	0.834	0.898	10.120	1.426
P21333	Filamin-A FLNA	18	2103	41	77	1.227	0.967	1.509	2.216	1.357
P22105	*Tenascin*-*X TNXB*	18	85	3	3	1.548	1.570	1.155	2.143	0.973
P04792	Heat shock protein beta-1 HSPB1	17.8	1495	8	69	1.298	0.775	1.077	4.369	1.586
Q12805	Protein EFEMP1	17.6	62	2	3	2.222	1.596	0.921	1.974	1.373
Q32P28	Prolyl 3-hydroxylase 1 LEPRE1	17.6	139	3	4	1.399	1.023	1.415	0.547	1.260
P21291	Cysteine and glycine-rich protein 1 CSRP1	17.4	489	6	17	0.912	1.039	1.080	21.887	0.735
P02452	*Collagen alpha*-*1(I) chain COL1A1*	17.4	830	11	45	1.124	0.428	0.904	5.938	2.536
P18206	Vinculin VCL	17.4	1911	29	73	1.404	0.904	1.131	2.429	1.425
P35749	Myosin-11 MYH11	17.2	1059	14	40	0.986	1.006	1.228	3.765	1.372
P12004	Proliferating cell nuclear antigen PCNA	17.2	107	4	7	0.954	0.709	1.790	0.414	1.326

Selection of proteins with biomarker score, protein score, numbers of unique peptides and peptide spectrum matches (PSMs) and ratios of expression for the different tissue comparisons. Proteins in italics are of particular note with several selected for serum testing

Protein groups were scored for biomarker potential with carboxypeptidase M (CPM) the highest scoring, displaying increased endometrial expression in endometriosis versus both control groups and in both cycle phases (ES/CS = 1.62, ES/PS = 2.53, EP/CP = 2.45) (Table 3). Its expression was however lower in ectopic tissue (EcS/ES = 0.32). Similarly, progesterone receptor (PGR) was over-expressed in endometriosis eutopic tissue (ES/CS = 1.45, ES/PS = 1.76, EP/CP = 2.42), whilst decreased in ectopic tissue (EcS/ES = 0.32). Membrane-associated progesterone receptor component 1 (PGRMC1) was similarly over-expressed in proliferative endometriosis eutopic tissue versus control (EP/CP = 1.56), and under-expressed in ectopic tissue (EcS/ES = 0.61). The progesterone-regulated gene glycodelin (PAEP) was also under-expressed in ectopic tissue (EcS/ES = 0.21). Other proteins of interest were tenascin C (TNC), up-regulated in endometriosis compared to both control groups (ES/CS = 1.82, ES/PS = 1.68, EP/CP = 1.50), and transgelin (TAGLN), increased in proliferative phase endometriosis versus controls (EP/CP = 2.17) and ectopic versus eutopic tissue (EcS/ES = 18.83). Notably, LUM and TPM2 were also identified with increased expression in ectopic tissue, similar to that observed by 2D-DIGE profiling, although were not altered appreciably in the eutopic tissue comparisons.

#### Testing candidates as serum biomarkers of endometriosis

From the tissue profiling, TNC, CPM, TPM2, LUM and PAEP were selected for further testing as serological markers using samples from 87 women (control n = 21; pain control n = 21; endometriosis n = 45) (Table 1). Biomarker candidates reported in the literature (sICAM1, MCP1, MIF, IL1R2, VEGF and FST) were also tested with CA125, progesterone, oestradiol and CRP. Commercial assays were first assessed using a test pool of all samples to determine optimal sample dilutions and reproducibility (Additional file 1: Table S1) and then run on the full set. Serum measurements were correlated with clinical group and cycle phase. Only CA125 and sICAM1 were significantly elevated (*P *< 0.05) when comparing endometriosis to both control groups considering all cycle phases (Table 4A). Areas under the ROC curve for the endometriosis versus pain groups were 0.713 for CA125 and 0.722 for sICAM1. TNC was significantly elevated in the pain group versus the control and endometriosis groups. In the secretory phase, CA125 elevation maintained significance when comparing endometriosis to both control groups, whereas sICAM1 was significantly raised only when comparing endometriosis and pain controls (Table 4B). Elevation of VEGF became significant between endometriosis and non-pain controls in secretory phase, whilst FST (lower in endometriosis) was the only candidate found to be significant when comparing groups in the proliferative phase. Together, these data suggest cycle dependency in the serum levels of some of the candidate markers.

**Table 4:** (A) Median concentrations and ranges of individual biomarker candidates in serum and *P* values for comparison of clinical groups in all stages of the menstrual cycle. (B) Analysis by menstrual cycle phase showing significant differences in candidate biomarker levels. *P* values < 0.05 are considered significant

Candidate biomarker	Units	Control (C)	Pain (P)	Endometriosis (E)	E versus C *P* value	E versus P *P* value	C versus P *P* value
(A)							
CA125	U/mL	7.7 (1.3–28.0)	7.7 (0.6–273.0)	22.5 (0.70–386.6)	0.0007	0.0058	ns
oestradiol	pmol/L	245 (18–2512)	419 (79–1616)	345 (18–2110)	ns	ns	ns
progesterone	nmol/L	1.9 (0.6–43.7)	6.1 (0.9–53.4)	2.3 (0.2–61.7)	ns	ns	ns
sICAM1	ng/mL	301.4 (81.9–502.3)	265.7 (85.83–624.7)	342.1 (92.3–730.8)	0.04	0.004	ns
IL1R2	ng/mL	12.72 (7.65–19.23)	11.33 (6.41–23.29)	12.35 (0.57–23.2)	ns	ns	ns
MCP1	ng/mL	0.32 (0.10–1.55)	0.26 (0.07–1.39)	0.27 (0.03–2.25)	ns	ns	ns
MIF	ng/mL	24.81 (2.20–106.3)	30.87 (2.22–282.8)	24.11 (2.25–314.6)	ns	ns	ns
VEGF	ng/mL	0.33 (0.07–1.00)	0.32(0.09–0.95)	0.41 (0.15–1.41)	ns	ns	ns
FST	ng/mL	0.82 (0.27–3.79)	0.73 (0.28–2.41)	0.67 (0.23–4.33)	ns	ns	ns
PAEP	ng/mL	7.22 (1.03–51.82)	11.35 (4.92–38.84)	13.52 (0.70–79.68)	ns	ns	ns
LUM	ng/mL	47.05 (0.60–101.1)	41.7 (0.18–111,3)	45.0 (6.39–133.0)	ns	ns	ns
TNC	ng/mL	40.31 (11.27–152.2)	73.24 (13.47–154.7)	46.27 (7.09–182.4)	ns	0.039	0.044
CPM	ng/mL	3.83 (0.4–70.75)	4.32(0.02–38.03)	6.13 (0.4–94.62)	ns	ns	ns
CRP	mg/L	0.7 (0.6–9.2)	0.6 (0.6–10.0)	0.75 (0.6–20.0)	ns	ns	ns

Candidate biomarker	Secretory phase		Proliferative phase	
	E (n = 26) versus C (n = 9)	E (n = 26) versus P (n = 12)	E (n = 13) versus C (n = 10)	E (n = 13) versus P (n = 9)
(B)				
CA125	0.006	0.003	ns	ns
oestrogen	ns	ns	ns	ns
progesterone	ns	ns	ns	ns
sICAM1	ns	0.026	ns	ns
IL1R2	ns	ns	ns	ns
MCP1	ns	ns	ns	ns
MIF	ns	ns	ns	ns
VEGF	0.033	ns	ns	ns
FST	ns	ns	0.011	ns
PAEP	ns	ns	ns	ns
LUM	ns	ns	ns	ns
TNC	ns	0.037	ns	ns
CPM	ns	ns	ns	ns
CRP	ns	ns	ns	ns

C = no pain control; P = pain control; E = endometriosis; ns = not significant

Multi-marker logistic regression models were generated to assess if candidates would complement one another to improve classification. Generally, cross-validated models for discriminating the endometriosis and pain groups (all phases) performed similarly to those discriminating the endometriosis and non-pain groups with sensitivities of 62–67% at 80% specificity (Table 5). sICAM1 featured in the best models for both groups, whilst CA125 was only included in models for discriminating endometriosis from the non-pain group. The best performing model [sICAM1, FST, TNC] for discriminating endometriosis from pain controls (E vs. P; all phases), gave a sensitivity of 67% at 80% specificity. When considering menstrual phase, for which both control groups were pooled, the best model [ICAM1, FST, oestradiol] gave 77% sensitivity at 80% specificity for detecting endometriosis in the proliferative phase, whilst the best model for secretory phase samples [CA125, MIF, PAEP], gave a sensitivity of 65% at 80% specificity. This again highlights cycle-dependency in the performance of the biomarker panels.

**Table 5:** Performance of cross-validated multi-marker models for discriminating endometriosis from control groups

Models	AUC	Sensitivity	Specificity
E versus C (all phases)			
CA125, sICAM1, CPM	0.768	0.667	0.8
CA125, sICAM1, VEGF	0.777	0.644	0.8
CA125, sICAM1, FST	0.77	0.644	0.8
CA125, sICAM1	0.778	0.6	0.9
CA125, sICAM1, IL1R2	0.758	0.6	0.9
CA125, sICAM1, MCP1	0.757	0.6	0.9
E versus P (all phases)			
sICAM1, FST, TNC	0.679	0.667	0.8
sICAM1, TNC	0.708	0.622	0.8
sICAM1, TNC, Oestradiol	0.68	0.622	0.8
sICAM1, PAEP, TNC	0.695	0.622	0.8
sICAM1, MIF, PAEP	0.697	0.622	0.8
sICAM1, LUM	0.665	0.444	0.9
E versus C + P (all phases)			
CA125, sICAM1, FST, CPM	0.706	0.578	0.8
CA125, sICAM1, VEGF, PAEP	0.71	0.578	0.8
CA125, sICAM1, PAEP	0.719	0.578	0.8
CA125, sICAM1, MIF, PAEP	0.704	0.578	0.8
CA125, MIF	0.621	0.467	0.9
E versus C + P (Proliferative)			
sICAM1, FST, Oestradiol	0.769	0.769	0.8
sICAM1, MIF, FST	0.781	0.692	0.8
sICAM1, FST	0.802	0.692	0.8
CRP, sICAM1, FST	0.802	0.692	0.8
CA125, sICAM1, FST	0.814	0.692	0.8
sICAM1, MIF, FST	0.781	0.615	0.9
E versus C + P (Secretory)			
CA125, MIF, PAEP	0.705	0.654	0.8
CA125, sICAM1, MIF	0.725	0.615	0.8
CA125, MIF, TNC	0.683	0.615	0.8
CA125, MIF, PAEP	0.705	0.538	0.9
CA125, MIF, TNC	0.683	0.577	0.9

Models were generated by logistic regression using up to 4 candidates with cross-validation by leave-one-out. The best performing models (by sensitivity) and area under the ROC curve (AUC) are reported for each comparison at fixed specificities of 0.90 or 0.80. E = endometriosis; C = no pain controls; P pain controls. Control groups were pooled (C + P) for some of the analyses

## Discussion

The aim of this study was a tissue-based discovery of potential new biomarkers of endometriosis with translation to serum-based tests aimed at non-invasive diagnosis. Promising biomarkers reported in the literature were also assessed. The main challenges experienced in the discovery was the heterogeneity of tissue samples and variable blood contamination. This was evidenced by the presence of variable levels of abundant fibro-muscular and serum proteins across the sample set that will have compromised the quality of the analysis. Whilst pooling would average out some of this heterogeneity, it is possible that outlier samples may skew the data leading to a higher false discovery rate. Despite this, a number of differentially expressed proteins were identified as potential biomarkers that were assayed in serum samples and which contributed to multivariate models that could discriminate endometriosis from either or both control groups with reasonable accuracy.

Notably, we did not find CA125 or any of the previously reported candidate biomarkers using the proteomic approaches described. CA125 exists at relatively low abundance and is an extremely large (up to 4 MDa), heterogeneously glycosylated protein. These properties would make sampling of CA125 using these methods unlikely—the protein is not likely to resolve well on 2D gels due to its large size and heterogeneity, whilst its heavy modification may hinder efficient tryptic digestion and decrease mass matching in database searches. Similarly, we postulate that the other candidates (sICAM1, MCP1, MIF, IL1R2, VEGF and FST) exist at relatively low abundance in tissue, reducing the chance of their identification. This highlights that coverage of the proteome is still somewhat limited despite the multi-dimensional approach used in our methodology.

Tissue profiling identified numerous differentially expressed proteins implicated in the implantation of endometriotic tissue beyond the endometrium and/or involved in disease progression. Although the enrichment analysis was somewhat ambiguous, multiple proteins involved in cytoskeletal and ECM organisation and cell–matrix interactions were enriched. This supports findings from previous studies [11–14]. Adhesion to the peritoneal ECM and invasion of retrograde-shed endometrial cells is one of the vital stages in implantation of ectopic endometrial cells and it is tempting to speculate that the adhesion/ECM proteins identified herein play important roles in this process. Lumican (LUM) has a role in cell migration and proliferation during embryonic development, tissue repair and tumour growth through regulation of matrix metalloprotease activity [15] and collagen fibrillogenesis [16]. In the endometrium, LUM expression was reported to increase in the secretory phase [17], in agreement with our findings. We also found multiple collagens and other ECM glycoproteins and integrin ligands to be overexpressed in endometriotic samples, and particularly in ectopic versus eutopic tissue. One such protein, tenascin C (TNC), was reported to be regulated across the menstrual cycle and aberrantly in endometriosis [18–20]. Our data suggest that its cyclic expression is lost in the endometrium of endometriosis patients, and whilst we did not observe TNC overexpression in ectopic lesions, its increased expression may promote adhesion and invasion of endometrial cells at ectopic sites.

Tropomyosins TPM1 and TPM2 play a role in muscle contraction, motility, maintenance of cell shape and cell–matrix interactions through stabilisation of actin filaments. Expression of TPM2 was higher in ectopic versus eutopic tissue and increased in secretory phase eutopic tissue from endometriosis patients compared to both control groups. This may point to its involvement in mediating cellular structural changes that allow movement and invasion of endometrial cells. The similarly expressed smooth muscle actin-binding protein TAGLN, may also be involved in this process and supports previous findings [21]. Increased expression of TPM1, TPM2 and TAGLN may be the result of a higher smooth muscle content of deep endometriotic lesions. Another selected candidate was CPM; a GPI-anchored extracellular peptidase that functions in processing of peptide hormones, chemokines and growth factors. It has a reported role in inflammation and stem cell mobilisation and has been shown to be up-regulated in endometrial epithelial cells during the proliferative phase [22]. The increased expression in endometriosis observed herein, may support the inflammatory response, although its lower expression in ectopic lesions is somewhat at odds with this.

Of particular note was the differential expression of the progesterone receptor (PGR). Several genes found to be dysregulated in the endometrium of endometriosis patients are known progesterone targets (*Foxo1a*, *Mig6* and *Cyp26a1*) and their overall pattern of expression suggested prolongation of the proliferative phase [23]. Indeed, incomplete transition of the endometrium from the proliferative to the secretory phase is a hallmark of endometriosis that has been attributed to progesterone resistance [24]. Our finding of reduced PGR expression in ectopic tissue has been previously reported as a possible mechanism of progesterone resistance [25]. However, whether the observed higher expression of PGR observed in eutopic endometrium from endometriosis patients is involved in progesterone resistance, or a response to it, is unclear. In part agreement with the literature [26–28], we also showed reduced expression of PGRMC1 and the progesterone target gene PAEP in endometriotic lesions; changes that may also contribute to progesterone resistance in ectopic lesions.

CA125 was identified as the best single marker for discriminating endometriosis from controls and featured prominently in the best-performing multimarker models. It is noteworthy that CA125 is currently the best single serum marker for ovarian cancer, although its median level in the endometriosis samples fell below the clinical threshold of 35 U/mL used for ovarian cancer detection. CA125 has been investigated extensively as a circulating marker of endometriosis, although lacks diagnostic accuracy when used alone. Our data supports this notion, with serum CA125 giving 40% sensitivity at 90% specificity in our cohort. Its performance was also cycle-dependent, being better at discriminating secretory phase samples. Cyclic differences in CA125 levels in endometriosis have been reported previously [29–32], although the diagnostic benefit of taking cycle stage into account is unclear. Soluble ICAM1 has also been investigated as a circulating marker with conflicting reports on its usefulness as a biomarker [33–37]. Our data do not support its use alone as a diagnostic marker, however its inclusion in our best classification models, suggests its value when combined with other markers, particularly CA125, FST and TNC.

Previous studies have tested panels of serum or plasma biomarkers, including those investigated here. Kocbek et al. reported a model using the ratio of leptin to glycodelin/PAEP and age, with 83.6% sensitivity and 83.8% specificity for distinguishing ovarian endometriosis from controls independently of cycle phase [38]. Although also tested in this study, ICAM1, VEGF, CRP and MCP1 were not included in the reported best models, whilst CA125 was not assessed. In another study, 28 plasma proteins were assessed in a large patient cohort, with an independently validated model comprising CA125, annexin V, VEGF and sICAM1/or PAEP giving 81–90% sensitivity and 63–81% specificity for detecting endometriosis in the menstrual phase [39]. Other reported models including CA125, MCP1 and/or MIF showed reasonable diagnostic accuracies [40, 41]. Whilst MCP1 added little to our models, MIF was in the best models for discriminating endometriosis from both control groups, particularly for the secretory phase. Our data thus corroborate CA125, sICAM1, PAEP, MIF and FST as potentially useful diagnostic markers when combined in multivariate models.

## Conclusions

In conclusion, we have identified molecular changes associated with endometriosis in eutopic and ectopic tissue and have derived non-invasive, cycle phase-specific diagnostic models for endometriosis with respectable performance characteristics that are similar, if not better, than those reported previously. Our study has some weaknesses. Firstly, models were only tested by cross-validation on the same dataset with some evidence of overfitting and subgrouping by cycle phase has underpowered the study. Thus, validation of these models in a larger independent cohort is necessary and should include samples from patients with other gynaecological conditions presenting with similar symptoms, particularly gynaecological malignancies. This would allow better assessment of the specificity of the biomarker panels. Secondly, our best biomarker model for discriminating endometriosis from the more relevant pain control group [sICAM1, FST, TNC] provided a sensitivity of 67% at 80% specificity, and so its usefulness as a triage test for guiding surgery is debatable. However, taking cycle phase into account, one model provided 61.5% sensitivity at 90% specificity, and so might be acceptable for triaging. Thirdly, whilst we show improved model performances by taking cycle-phase into account (particularly for proliferative phase), this type of testing may be difficult to implement in clinical practice should the models be validated.

## Additional files

**Additional file 1: Table S1.** ELISA assays used with optimal dilutions and intra-assay CVs. **Table S2.** List of differentially expressed protein identifications from 2D-DIGE profiling. **Figure S1.** Quality control of tissue lysates and immunodepletion test. **Figure S2.** Overlaid fluorescent 2D gel images from main 2D-DIGE profiling experiment.

**Additional file 2.** Results of TMT-based LC-MS/MS profiling with candidate scoring.

## Abbreviations

2D-DIGE: 2D-difference gel electrophoresis
TMT–LC–MS/MS: tandem mass tagging–liquid chromatography–tandem mass spectrometry
AUC: area under the receiver operating characteristic curve
DTT: dithiothreitol
IAM: iodoacetamide
LC–MS/MS: liquid chromatography–tandem mass spectrometry
ACN: acetonitrile
AmBiC: ammonium bicarbonate
FA: formic acid
RPLC: reversed phase-liquid chromatography
SAX: strong anion exchange
TEAB: triethylammonium bicarbonate
