# Supplementary material for: Discovery of non-invasive biomarkers for the diagnosis of endometriosis
Source: Clin Proteomics. 2019 Apr 6;16:14. doi: 10.1186/s12014-019-9235-3 (PMC6451201; doi:10.1186/s12014-019-9235-3)
Supplement: Supplementary file 1 — Additional file 1: Table S1. ELISA assays used with optimal dilutions and intra-assay CVs. Table S2. List of differentially expressed protein identifications from 2D-DIGE profiling. Figure S1. Quality control of tissue lysates and immunodepletion test. Figure S2. Overlaid fluorescent 2D gel images from main 2D-DIGE profiling experiment. [file 12014_2019_9235_MOESM1_ESM.docx]

Table S1. ELISA assays, optimal dilutions and intra-assay CVs.

| **Assay** | **Source** | **Optimal dilution** | **Intra-assay CV%** |
| --- | --- | --- | --- |
| CPM | Clone Corp | 1:20 | 5.0 |
| FST | R&D | 1:2 | 5.6 |
| sICAM | R&D | 1:50 | 2.0 |
| PAEP | Bioserv | 1:5 | 13.6 |
| IL1R-II | R&D | 1:50 | 1.5 |
| LUM | Boster | 1:20 | 10.6 |
| MCP1 | R&D | 1:10 | 5.5 |
| MIF | R&D | 1:10 | 4.5 |
| TNC | Abcam | 1:50 | 0.7 |
| VEGF | R&D | 1:2 | 4.6 |
| CA125 | Roche | 1:1 | 4.0 |

**Table S2. List of differentially expressed protein identifications from 2D-DIGE profiling.** Protein identifications from gel spots are listed along with numbers of peptide matches, unique peptide sequences, predicted molecular weights and gel-based p*I* and molecular weights. Many of the spots yielded multiple protein identifications. Three spots yielded no identifications. Average protein expression ratios and *P* values are shown for the comparison of eutopic tissue from clinical groups ES (endometriosis secretory phase) *versus* CS (no pain controls secretory phase) and PS (pain controls secretory phase), EP (endometriosis proliferative phase) *versus* CP (no pain controls proliferative phase) and EcS (ectopic tissue endometriosis secretory phase) *versus* ES. Ratios in red show expression >1.5-fold and *P*<0.05, whilst those in blue were >-1.5-fold and *P*<0.05. A simple marker score was used to prioritise candidates for further testing and was based on proteins displaying the same direction of differential regulation between endometriosis and both control groups and in ectopic *versus* eutopic tissue. Proteins in yellow were selected for further testing in serum samples.

|  |  |  |  |  |  |  |  |  | **ES vs. CS** | | **ES vs. PS** | | **EP vs. CP** | | **EcS vs. ES** | |  |
| --- | --- | --- | --- | --- | --- | --- | --- | --- | --- | --- | --- | --- | --- | --- | --- | --- | --- |
| **Master Spot No.** | **Acc No.** | **Protein Name** | **Score** | **Peptide Matches** | **Peptide Sequences** | **Pred Mass** | **Gel pI** | **Gel Mass** | **Av. Ratio** | **T-test** | **Av. Ratio** | **T-test** | **Av. Ratio** | **T-test** | **Av. Ratio** | **T-test** | **Marker score** |
| 708 | P51884 | Lumican LUM | 394 | 15 | 7 | 38747 | 3.74 | 73000 | 1.86 | 0.0051 | 2.25 | 0.0043 | 1.63 | 0.1600 | 2.09 | 0.0083 | 4 |
| 708 | P01011 | Alpha-1-antichymotrypsin SERPINA3 | 69 | 2 | 2 | 47792 | 3.74 | 73000 | 1.86 | 0.0051 | 2.25 | 0.0043 | 1.63 | 0.1600 | 2.09 | 0.0083 | 4 |
| 833 | P51884 | Lumican LUM | 314 | 14 | 7 | 38747 | 3.87 | 65000 | 2.05 | 0.0250 | 2.36 | 0.0290 | 1.04 | 0.7800 | 1.80 | 0.0064 | 4 |
| 833 | P08670 | Vimentin VIM | 92 | 5 | 4 | 53676 | 3.87 | 65000 | 2.05 | 0.0250 | 2.36 | 0.0290 | 1.04 | 0.7800 | 1.80 | 0.0064 | 4 |
| 833 | O95302 | Peptidyl-prolyl cis-trans isomerase FKBP9 | 114 | 5 | 3 | 63500 | 3.87 | 65000 | 2.05 | 0.0250 | 2.36 | 0.0290 | 1.04 | 0.7800 | 1.80 | 0.0064 | 4 |
| 833 | O75781 | Paralemmin-1 PALM | 97 | 2 | 2 | 42221 | 3.87 | 65000 | 2.05 | 0.0250 | 2.36 | 0.0290 | 1.04 | 0.7800 | 1.80 | 0.0064 | 4 |
| 1363 | O75874 | Isocitrate dehydrogenase [NADP] cytoplasmic IDH1 | 474 | 23 | 11 | 46915 | 6.59 | 41000 | -1.24 | 0.0110 | -1.97 | 0.0005 | -2.40 | 0.0009 | -3.39 | 0.0000 | 4 |
| 1463 |  | No identification |  |  |  |  | 8.38 | 37000 | -1.58 | 0.0067 | -1.43 | 0.0510 | -1.61 | 0.0490 | -1.90 | 0.0057 | 4 |
| 1543 | P07951 | Tropomyosin beta chain TPM2 | 1066 | 37 | 12 | 32945 | 3.5 | 33000 | 3.17 | 0.0010 | 2.13 | 0.0039 | 1.10 | 0.1100 | 4.65 | 0.0014 | 4 |
| 1543 | P08670 | Vimentin VIM | 376 | 15 | 10 | 53676 | 3.5 | 33000 | 3.17 | 0.0010 | 2.13 | 0.0039 | 1.10 | 0.1100 | 4.65 | 0.0014 | 4 |
| 1548 | P07951 | Tropomyosin beta chain TPM2 | 885 | 33 | 10 | 32945 | 3.77 | 30000 | 3.15 | 0.0000 | 2.36 | 0.0057 | 1.15 | 0.1900 | 4.15 | 0.0002 | 4 |
| 1548 | P08670 | Vimentin VIM | 268 | 15 | 11 | 53676 | 3.77 | 30000 | 3.15 | 0.0000 | 2.36 | 0.0057 | 1.15 | 0.1900 | 4.15 | 0.0002 | 4 |
| 1548 | P51858 | Hepatoma-derived growth factor HDGF | 77 | 3 | 2 | 26886 | 3.77 | 30000 | 3.15 | 0.0000 | 2.36 | 0.0057 | 1.15 | 0.1900 | 4.15 | 0.0002 | 4 |
| 1689 | Q32P51 | Heterogeneous nuclear ribonucleoprotein A1-like 2 HNRNPA1L2 | 113 | 2 | 2 | 34375 | 9.56 | 24000 | -1.43 | 0.0460 | -1.48 | 0.0310 | -1.34 | 0.0300 | -5.71 | 0.0002 | 4 |
| 1981 | P02647 | Apolipoprotein A-I APOA1 | 1190 | 40 | 16 | 30759 | 4.2 | 15000 | 1.70 | 0.0650 | 3.07 | 0.0065 | 1.43 | 0.0670 | 1.59 | 0.1300 | 4 |
| 1981 | P12111 | Collagen alpha-3(VI)chain COL6A3 | 206 | 9 | 7 | 345167 | 4.2 | 15000 | 1.70 | 0.0650 | 3.07 | 0.0065 | 1.43 | 0.0670 | 1.59 | 0.1300 | 4 |
| 1981 | P60709 | Actin, cytoplasmic 1 ACTB | 244 | 7 | 6 | 42052 | 4.2 | 15000 | 1.70 | 0.0650 | 3.07 | 0.0065 | 1.43 | 0.0670 | 1.59 | 0.1300 | 4 |
| 1981 | P04792 | Heat shock protein beta-1 HSPB1 | 101 | 6 | 4 | 22826 | 4.2 | 15000 | 1.70 | 0.0650 | 3.07 | 0.0065 | 1.43 | 0.0670 | 1.59 | 0.1300 | 4 |
| 492 | P06396 | Gelsolin GSN | 195 | 10 | 4 | 86043 | 5.17 | 88000 | -1.02 | 0.6900 | -1.56 | 0.0052 | -1.92 | 0.0003 | -2.92 | 0.0043 | 3 |
| 492 | P21333 | Filamin-A FLNA | 149 | 6 | 6 | 283301 | 5.17 | 88000 | -1.02 | 0.6900 | -1.56 | 0.0052 | -1.92 | 0.0003 | -2.92 | 0.0043 | 3 |
| 492 | P07900 | Heat shock protein HSP 90-alpha HSP90AA1 | 148 | 5 | 5 | 85006 | 5.17 | 88000 | -1.02 | 0.6900 | -1.56 | 0.0052 | -1.92 | 0.0003 | -2.92 | 0.0043 | 3 |
| 492 | O75369 | Filamin-B FLNB | 105 | 4 | 4 | 280157 | 5.17 | 88000 | -1.02 | 0.6900 | -1.56 | 0.0052 | -1.92 | 0.0003 | -2.92 | 0.0043 | 3 |
| 497 | P07900 | Heat shock protein HSP 90-alpha HSP90AA1 | 103 | 4 | 3 | 85006 | 5.7 | 90000 | -1.04 | 0.6700 | -1.63 | 0.0160 | -1.55 | 0.0004 | -2.17 | 0.0130 | 3 |
| 604 | P11021 | 78 kDa glucose-regulated protein HSPA5 | 3062 | 99 | 34 | 72402 | 4.09 | 80000 | -1.20 | 0.0610 | -1.74 | 0.0027 | -1.62 | 0.0022 | -9.47 | 0.0008 | 3 |
| 604 | P51884 | Lumican LUM | 101 | 4 | 2 | 38747 | 4.09 | 80000 | -1.20 | 0.0610 | -1.74 | 0.0027 | -1.62 | 0.0022 | -9.47 | 0.0008 | 3 |
| 604 | P14923 | Junction plakoglobin JUP | 79 | 3 | 3 | 82434 | 4.09 | 80000 | -1.20 | 0.0610 | -1.74 | 0.0027 | -1.62 | 0.0022 | -9.47 | 0.0008 | 3 |
| 883 | O60701 | UDP-glucose 6-dehydrogenase UGDH | 753 | 29 | 14 | 55674 | 7.52 | 65000 | -1.53 | 0.0002 | -1.53 | 0.0001 | 1.02 | 0.8400 | -1.46 | 0.2100 | 3 |
| 883 | P14866 | Heterogeneous nuclear ribonucleoprotein L HNRNPL | 575 | 20 | 6 | 64720 | 7.52 | 65000 | -1.53 | 0.0002 | -1.53 | 0.0001 | 1.02 | 0.8400 | -1.46 | 0.2100 | 3 |
| 883 | P04040 | Catalase CAT | 242 | 13 | 8 | 59947 | 7.52 | 65000 | -1.53 | 0.0002 | -1.53 | 0.0001 | 1.02 | 0.8400 | -1.46 | 0.2100 | 3 |
| 883 | P30038 | Delta-1-pyrroline-5-carboxylate dehydrogenase, mitochondrial ALDH4A1 | 91 | 4 | 3 | 62137 | 7.52 | 65000 | -1.53 | 0.0002 | -1.53 | 0.0001 | 1.02 | 0.8400 | -1.46 | 0.2100 | 3 |
| 962 | P78371 | T-complex protein 1 subunit beta CCT2 | 169 | 3 | 2 | 57794 | 5.9 | 59000 | 1.25 | 0.0130 | 1.52 | 0.0100 | 1.55 | 0.0220 | 2.56 | 0.0030 | 3 |
| 1149 | P31943 | Heterogeneous nuclear ribonucleoprotein H HNRNPH1 | 827 | 28 | 10 | 49484 | 5.34 | 51000 | -1.21 | 0.2600 | -1.64 | 0.0330 | -1.77 | 0.0000 | -2.08 | 0.0360 | 3 |
| 1149 | P05091 | Aldehyde dehydrogenase, mitochondrial ALDH2 | 105 | 5 | 4 | 56859 | 5.34 | 51000 | -1.21 | 0.2600 | -1.64 | 0.0330 | -1.77 | 0.0000 | -2.08 | 0.0360 | 3 |
| 1210 | P61158 | Actin-related protein 3 ACTR3 | 119 | 4 | 4 | 47797 | 5.8 | 50000 | 1.02 | 0.9900 | -1.73 | 0.0180 | -4.03 | 0.0047 | -1.93 | 0.0270 | 3 |
| 1210 | P36957 | Dihydrolipoyllysine-residue succinyltransferase component of 2-oxoglutarate dehydrogenase complex, mitochondrial DLST | 139 | 4 | 4 | 49067 | 5.8 | 50000 | 1.02 | 0.9900 | -1.73 | 0.0180 | -4.03 | 0.0047 | -1.93 | 0.0270 | 3 |
| 1210 | P35998 | 26S protease regulatory subunit 7 PSMC2 | 66 | 3 | 3 | 49002 | 5.8 | 50000 | 1.02 | 0.9900 | -1.73 | 0.0180 | -4.03 | 0.0047 | -1.93 | 0.0270 | 3 |
| 1210 | Q9BQE3 | Tubulin alpha-1C chain TUBA1C | 47 | 2 | 2 | 50548 | 5.8 | 50000 | 1.02 | 0.9900 | -1.73 | 0.0180 | -4.03 | 0.0047 | -1.93 | 0.0270 | 3 |
| 1212 | Q5VTE0 | Putative elongation factor 1-alpha-like 3 EEF1A1P5 PE=5 | 365 | 17 | 9 | 50495 | 9 | 46000 | -1.14 | 0.2600 | -1.76 | 0.0083 | -2.11 | 0.0061 | -8.86 | 0.0002 | 3 |
| 1368 | O75874 | Isocitrate dehydrogenase [NADP] cytoplasmic IDH1 | 916 | 39 | 17 | 46915 | 7.5 | 42000 | -1.33 | 0.0027 | -2.15 | 0.0001 | -3.09 | 0.0000 | -4.11 | 0.0000 | 3 |
| 1370 | P00558 | Phosphoglycerate kinase 1 PGK1 | 1016 | 38 | 19 | 44985 | 7.7 | 42000 | -1.47 | 0.0000 | -1.57 | 0.0000 | 1.07 | 0.6900 | -1.61 | 0.1000 | 3 |
| 1370 | P62333 | 26S protease regulatory subunit 10B PSMC6 | 73 | 3 | 3 | 44430 | 7.7 | 42000 | -1.47 | 0.0000 | -1.57 | 0.0000 | 1.07 | 0.6900 | -1.61 | 0.1000 | 3 |
| 1371 | Q9UBG3 | Cornulin CRNN | 205 | 8 | 4 | 53730 | 7.24 | 41000 | -1.33 | 0.0032 | -2.33 | 0.0002 | -4.01 | 0.0000 | -3.98 | 0.0002 | 3 |
| 1371 | P04083 | Annexin A1 ANXA1 | 314 | 7 | 6 | 38918 | 7.24 | 41000 | -1.33 | 0.0032 | -2.33 | 0.0002 | -4.01 | 0.0000 | -3.98 | 0.0002 | 3 |
| 1666 | P04083 | Annexin A1 ANXA1 | 1136 | 34 | 16 | 38918 | 6.87 | 25000 | -1.05 | 0.2400 | -1.72 | 0.0001 | -1.69 | 0.0000 | -2.97 | 0.0000 | 3 |
| 1666 | Q9H9H4 | Vacuolar protein sorting-associated protein 37B VPS37B | 75 | 3 | 2 | 31345 | 6.87 | 25000 | -1.05 | 0.2400 | -1.72 | 0.0001 | -1.69 | 0.0000 | -2.97 | 0.0000 | 3 |
| 1666 | O00151 | PDZ and LIM domain protein 1 PDLIM1 | 52 | 2 | 2 | 36505 | 6.87 | 25000 | -1.05 | 0.2400 | -1.72 | 0.0001 | -1.69 | 0.0000 | -2.97 | 0.0000 | 3 |
| 1997 | P01834 | Ig kappa chain C region IGKC | 104 | 4 | 2 | 11773 | 4.2 | 15000 | 1.10 | 0.1400 | 1.85 | 0.0004 | 1.44 | 0.0019 | 2.15 | 0.0011 | 3 |
| 1997 | P01593 | Ig kappa chain V-I region AG | 118 | 3 | 2 | 12099 | 4.2 | 15000 | 1.10 | 0.1400 | 1.85 | 0.0004 | 1.44 | 0.0019 | 2.15 | 0.0011 | 3 |
| 403 | P55072 | Transitional endoplasmic reticulum ATPase VCP | 175 | 6 | 3 | 89950 | 4 | 100000 | -1.22 | 0.0270 | -1.57 | 0.0065 | -1.15 | 0.0420 | -2.75 | 0.0000 | 2 |
| 404 | P55072 | Transitional endoplasmic reticulum ATPase VCP | 1177 | 36 | 16 | 89950 | 4.34 | 95000 | -1.17 | 0.0008 | -1.68 | 0.0054 | -1.20 | 0.0180 | -2.16 | 0.0082 | 2 |
| 745 | P54652 | Heat shock-related 70 kDa protein 2 HSPA2 | 603 | 25 | 13 | 70263 | 5 | 67000 | 1.64 | 0.0370 | 1.52 | 0.1800 | 1.21 | 0.2500 | -1.18 | 0.3700 | 2 |
| 745 | P38646 | Stress-70 protein, mitochondrial HSPA9 | 105 | 4 | 3 | 73920 | 5 | 67000 | 1.64 | 0.0370 | 1.52 | 0.1800 | 1.21 | 0.2500 | -1.18 | 0.3700 | 2 |
| 754 | P02545 | Prelamin-A/C LMNA | 927 | 38 | 19 | 74380 | 7 | 65000 | -1.12 | 0.2300 | -1.60 | 0.0160 | -1.25 | 0.0440 | -1.90 | 0.0047 | 2 |
| 754 | Q9NSD9 | Phenylalanine--tRNA ligase beta subunit FARSB | 173 | 8 | 6 | 66701 | 7 | 65000 | -1.12 | 0.2300 | -1.60 | 0.0160 | -1.25 | 0.0440 | -1.90 | 0.0047 | 2 |
| 754 | Q9Y3Z3 | Deoxynucleoside triphosphate triphosphohydrolase SAMHD1 | 94 | 6 | 6 | 72896 | 7 | 65000 | -1.12 | 0.2300 | -1.60 | 0.0160 | -1.25 | 0.0440 | -1.90 | 0.0047 | 2 |
| 754 | P51888 | Prolargin PRELP | 167 | 6 | 4 | 44181 | 7 | 65000 | -1.12 | 0.2300 | -1.60 | 0.0160 | -1.25 | 0.0440 | -1.90 | 0.0047 | 2 |
| 767 | Q02413 | Desmoglein-1 DSG1 | 91 | 4 | 4 | 114702 | 7.31 | 70000 | -1.12 | 0.0680 | -1.80 | 0.0260 | -1.39 | 0.0620 | -1.92 | 0.0006 | 2 |
| 767 | P02545 | Prelamin-A/C LMNA | 101 | 3 | 3 | 74380 | 7.31 | 70000 | -1.12 | 0.0680 | -1.80 | 0.0260 | -1.39 | 0.0620 | -1.92 | 0.0006 | 2 |
| 864 | P15924 | Desmoplakin DSP | 604 | 20 | 16 | 334021 | 4.43 | 65000 | -1.11 | 0.2800 | -1.53 | 0.0200 | -1.37 | 0.0016 | -2.45 | 0.0280 | 2 |
| 864 | P10809 | 60 kDa heat shock protein, mitochondrial HSPD1 | 866 | 17 | 9 | 61187 | 4.43 | 65000 | -1.11 | 0.2800 | -1.53 | 0.0200 | -1.37 | 0.0016 | -2.45 | 0.0280 | 2 |
| 864 | P07339 | Cathepsin D CTSD | 291 | 8 | 7 | 45037 | 4.43 | 65000 | -1.11 | 0.2800 | -1.53 | 0.0200 | -1.37 | 0.0016 | -2.45 | 0.0280 | 2 |
| 864 | P07355 | Annexin A2 ANXA2 | 154 | 4 | 4 | 38808 | 4.43 | 65000 | -1.11 | 0.2800 | -1.53 | 0.0200 | -1.37 | 0.0016 | -2.45 | 0.0280 | 2 |
| 864 | Q13867 | Bleomycin hydrolase BLMH | 76 | 3 | 2 | 53155 | 4.43 | 65000 | -1.11 | 0.2800 | -1.53 | 0.0200 | -1.37 | 0.0016 | -2.45 | 0.0280 | 2 |
| 864 | Q96QA5 | Gasdermin-A GSDMA | 78 | 3 | 2 | 49619 | 4.43 | 65000 | -1.11 | 0.2800 | -1.53 | 0.0200 | -1.37 | 0.0016 | -2.45 | 0.0280 | 2 |
| 864 | P04040 | Catalase CAT | 70 | 2 | 2 | 59947 | 4.43 | 65000 | -1.11 | 0.2800 | -1.53 | 0.0200 | -1.37 | 0.0016 | -2.45 | 0.0280 | 2 |
| 899 | P04040 | Catalase CAT | 1337 | 42 | 16 | 59947 | 7.64 | 61000 | -1.64 | 0.0017 | -1.82 | 0.0013 | 1.11 | 0.1100 | -1.37 | 0.1300 | 2 |
| 899 | P14618 | Pyruvate kinase PKM PKM | 506 | 15 | 8 | 58470 | 7.64 | 61000 | -1.64 | 0.0017 | -1.82 | 0.0013 | 1.11 | 0.1100 | -1.37 | 0.1300 | 2 |
| 899 | Q99832 | T-complex protein 1 subunit eta CCT7 | 45 | 2 | 2 | 59842 | 7.64 | 61000 | -1.64 | 0.0017 | -1.82 | 0.0013 | 1.11 | 0.1100 | -1.37 | 0.1300 | 2 |
| 899 | O60701 | UDP-glucose 6-dehydrogenase UGDH | 82 | 2 | 2 | 55674 | 7.64 | 61000 | -1.64 | 0.0017 | -1.82 | 0.0013 | 1.11 | 0.1100 | -1.37 | 0.1300 | 2 |
| 998 | P50995 | Annexin A11 ANXA11 | 186 | 6 | 5 | 54697 | 8.04 | 57000 | -1.32 | 0.0460 | -1.89 | 0.0072 | -1.28 | 0.1200 | -2.43 | 0.0400 | 2 |
| 998 | P00390 | Glutathione reductase, mitochondrial GSR | 132 | 5 | 4 | 56791 | 8.04 | 57000 | -1.32 | 0.0460 | -1.89 | 0.0072 | -1.28 | 0.1200 | -2.43 | 0.0400 | 2 |
| 998 | P34897 | Serine hydroxymethyltransferase, mitochondrial SHMT2 | 59 | 2 | 2 | 56414 | 8.04 | 57000 | -1.32 | 0.0460 | -1.89 | 0.0072 | -1.28 | 0.1200 | -2.43 | 0.0400 | 2 |
| 1150 | P25705 | ATP synthase subunit alpha, mitochondrial ATP5A1 | 402 | 14 | 7 | 59828 | 8.47 | 51000 | -1.57 | 0.0410 | 1.04 | 0.9100 | -1.25 | 0.0290 | -1.80 | 0.0240 | 2 |
| 1150 | P14618 | Pyruvate kinase PKM PKM | 39 | 2 | 2 | 58470 | 8.47 | 51000 | -1.57 | 0.0410 | 1.04 | 0.9100 | -1.25 | 0.0290 | -1.80 | 0.0240 | 2 |
| 1213 | Q5VTE0 | Putative elongation factor 1-alpha-like 3 EEF1A1P5 | 256 | 9 | 5 | 50495 | 9.41 | 48000 | -1.11 | 0.2800 | -1.68 | 0.0110 | -1.34 | 0.0340 | -9.97 | 0.0001 | 2 |
| 1234 | P17661 | Desmin DES | 428 | 15 | 11 | 53560 | 4.29 | 47000 | -1.54 | 0.0170 | 1.69 | 0.0360 | 1.65 | 0.0420 | 2.34 | 0.0090 | 2 |
| 1234 | P60709 | Actin, cytoplasmic 1 ACTB | 552 | 11 | 6 | 42052 | 4.29 | 47000 | -1.54 | 0.0170 | 1.69 | 0.0360 | 1.65 | 0.0420 | 2.34 | 0.0090 | 2 |
| 1234 | P01009 | Alpha-1-antitrypsin SERPINA1 | 449 | 9 | 5 | 46878 | 4.29 | 47000 | -1.54 | 0.0170 | 1.69 | 0.0360 | 1.65 | 0.0420 | 2.34 | 0.0090 | 2 |
| 1234 | P07437 | Tubulin beta chain TUBB | 205 | 7 | 6 | 50095 | 4.29 | 47000 | -1.54 | 0.0170 | 1.69 | 0.0360 | 1.65 | 0.0420 | 2.34 | 0.0090 | 2 |
| 1234 | O60664 | Perilipin-3 PLIN3 | 119 | 3 | 2 | 47217 | 4.29 | 47000 | -1.54 | 0.0170 | 1.69 | 0.0360 | 1.65 | 0.0420 | 2.34 | 0.0090 | 2 |
| 1234 | Q8TBC4 | NEDD8-activating enzyme E1 catalytic subunit UBA3 | 50 | 2 | 2 | 52504 | 4.29 | 47000 | -1.54 | 0.0170 | 1.69 | 0.0360 | 1.65 | 0.0420 | 2.34 | 0.0090 | 2 |
| 1270 | P06733 | Alpha-enolase ENO1 | 2245 | 68 | 20 | 47481 | 7.14 | 46000 | -1.20 | 0.0400 | -1.79 | 0.0001 | -1.18 | 0.0830 | -2.40 | 0.0004 | 2 |
| 1270 | Q9NVA2 | Septin-11 SEPT11 | 205 | 7 | 4 | 49652 | 7.14 | 46000 | -1.20 | 0.0400 | -1.79 | 0.0001 | -1.18 | 0.0830 | -2.40 | 0.0004 | 2 |
| 1319 | P49411 | Elongation factor Tu, mitochondrial TUFM | 1156 | 30 | 18 | 49852 | 7.14 | 43000 | -1.27 | 0.0110 | -1.55 | 0.0001 | -1.21 | 0.0470 | -2.92 | 0.0007 | 2 |
| 1319 | P06733 | Alpha-enolase ENO1 | 741 | 14 | 8 | 47481 | 7.14 | 43000 | -1.27 | 0.0110 | -1.55 | 0.0001 | -1.21 | 0.0470 | -2.92 | 0.0007 | 2 |
| 1319 | Q02413 | Desmoglein-1 DSG1 | 216 | 7 | 7 | 114702 | 7.14 | 43000 | -1.27 | 0.0110 | -1.55 | 0.0001 | -1.21 | 0.0470 | -2.92 | 0.0007 | 2 |
| 1319 | P15924 | Desmoplakin DSP | 67 | 5 | 5 | 334021 | 7.14 | 43000 | -1.27 | 0.0110 | -1.55 | 0.0001 | -1.21 | 0.0470 | -2.92 | 0.0007 | 2 |
| 1319 | P14923 | Junction plakoglobin JUP | 132 | 3 | 3 | 82434 | 7.14 | 43000 | -1.27 | 0.0110 | -1.55 | 0.0001 | -1.21 | 0.0470 | -2.92 | 0.0007 | 2 |
| 1319 | Q08554 | Desmocollin-1 DSC1 | 64 | 2 | 2 | 101406 | 7.14 | 43000 | -1.27 | 0.0110 | -1.55 | 0.0001 | -1.21 | 0.0470 | -2.92 | 0.0007 | 2 |
| 1323 | P60709 | Actin, cytoplasmic 1 ACTB | 242 | 8 | 5 | 42052 | 4.8 | 42000 | -1.17 | 0.1600 | -1.71 | 0.0068 | -1.88 | 0.0024 | -1.06 | 0.6500 | 2 |
| 1323 | P14923 | Junction plakoglobin JUP | 61 | 3 | 3 | 82434 | 4.8 | 42000 | -1.17 | 0.1600 | -1.71 | 0.0068 | -1.88 | 0.0024 | -1.06 | 0.6500 | 2 |
| 1323 | P12277 | Creatine kinase B-type CKB | 231 | 3 | 2 | 42902 | 4.8 | 42000 | -1.17 | 0.1600 | -1.71 | 0.0068 | -1.88 | 0.0024 | -1.06 | 0.6500 | 2 |
| 1323 | Q14240 | Eukaryotic initiation factor 4A-II EIF4A2 | 64 | 3 | 2 | 46601 | 4.8 | 42000 | -1.17 | 0.1600 | -1.71 | 0.0068 | -1.88 | 0.0024 | -1.06 | 0.6500 | 2 |
| 1351 | P00558 | Phosphoglycerate kinase 1 PGK1 | 1922 | 58 | 19 | 44985 | 8.46 | 41000 | -1.24 | 0.0100 | -1.77 | 0.0110 | -1.18 | 0.0820 | -4.95 | 0.0002 | 2 |
| 1351 | P50454 | Serpin H1 SERPINH1 | 241 | 9 | 4 | 46525 | 8.46 | 41000 | -1.24 | 0.0100 | -1.77 | 0.0110 | -1.18 | 0.0820 | -4.95 | 0.0002 | 2 |
| 1351 | P22695 | Cytochrome b-c1 complex subunit 2, mitochondrial UQCRC2 | 62 | 3 | 2 | 48584 | 8.46 | 41000 | -1.24 | 0.0100 | -1.77 | 0.0110 | -1.18 | 0.0820 | -4.95 | 0.0002 | 2 |
| 1515 | P02675 | Fibrinogen beta chain FGB | 546 | 23 | 10 | 56577 | 4.75 | 33000 | -1.35 | 0.0520 | 2.26 | 0.0130 | -1.46 | 0.0100 | 1.52 | 0.0480 | 2 |
| 1515 | P60709 | Actin, cytoplasmic 1 ACTB | 288 | 9 | 7 | 42052 | 4.75 | 33000 | -1.35 | 0.0520 | 2.26 | 0.0130 | -1.46 | 0.0100 | 1.52 | 0.0480 | 2 |
| 1675 | P02647 | Apolipoprotein A-I APOA1 | 1592 | 49 | 19 | 30759 | 4.3 | 28000 | -1.21 | 0.0640 | -1.56 | 0.0025 | -1.19 | 0.0220 | -2.24 | 0.0096 | 2 |
| 1675 | Q15181 | Inorganic pyrophosphatase PPA1 | 222 | 4 | 3 | 33095 | 4.3 | 28000 | -1.21 | 0.0640 | -1.56 | 0.0025 | -1.19 | 0.0220 | -2.24 | 0.0096 | 2 |
| 1693 | P04406 | Glyceraldehyde-3-phosphate dehydrogenase GAPDH | 1331 | 39 | 15 | 36201 | 7.5 | 28000 | -1.21 | 0.0050 | -1.50 | 0.0007 | -1.15 | 0.0650 | -2.93 | 0.0001 | 2 |
| 1693 | P40926 | Malate dehydrogenase, mitochondrial MDH2 | 695 | 22 | 11 | 35937 | 7.5 | 28000 | -1.21 | 0.0050 | -1.50 | 0.0007 | -1.15 | 0.0650 | -2.93 | 0.0001 | 2 |
| 1693 | P22626 | Heterogeneous nuclear ribonucleoproteins A2/B1 HNRNPA2B1 | 400 | 17 | 9 | 37464 | 7.5 | 28000 | -1.21 | 0.0050 | -1.50 | 0.0007 | -1.15 | 0.0650 | -2.93 | 0.0001 | 2 |
| 1850 | P00915 | Carbonic anhydrase 1 CA1 | 1838 | 49 | 10 | 28909 | 7.41 | 18000 | -1.58 | 0.0009 | -1.43 | 0.0310 | -1.51 | 0.0007 | 1.24 | 0.0850 | 2 |
| 1850 | P25789 | Proteasome subunit alpha type-4 PSMA4 | 66 | 3 | 2 | 29750 | 7.41 | 18000 | -1.58 | 0.0009 | -1.43 | 0.0310 | -1.51 | 0.0007 | 1.24 | 0.0850 | 2 |
| 1850 | Q13126 | S-methyl-5~-thioadenosine phosphorylase MTAP | 126 | 3 | 2 | 31729 | 7.41 | 18000 | -1.58 | 0.0009 | -1.43 | 0.0310 | -1.51 | 0.0007 | 1.24 | 0.0850 | 2 |
| 1905 |  | No identification |  |  |  |  | 7.91 | 17000 | -1.63 | 0.0250 | -1.01 | 0.9100 | -2.32 | 0.0059 | 1.23 | 0.3100 | 2 |
| 1924 | P68871 | Hemagloblin subunit beta HBB | 1024 | 29 | 9 | 16102 | 8.25 | 16000 | -1.71 | 0.0140 | 1.00 | 0.9200 | -1.60 | 0.0600 | 1.41 | 0.0110 | 2 |
| 1924 | P69905 | Hemagloblin subunit alpha HBA1 | 290 | 13 | 5 | 15305 | 8.25 | 16000 | -1.71 | 0.0140 | 1.00 | 0.9200 | -1.60 | 0.0600 | 1.41 | 0.0110 | 2 |
| 1924 | P00915 | Carbonic anhydrase 1 CA1 | 96 | 4 | 2 | 28909 | 8.25 | 16000 | -1.71 | 0.0140 | 1.00 | 0.9200 | -1.60 | 0.0600 | 1.41 | 0.0110 | 2 |
| 1929 | P68871 | Hemagloblin subunit beta HBB | 1564 | 41 | 11 | 16102 | 8.05 | 16000 | -1.92 | 0.0005 | -1.15 | 0.1300 | -1.85 | 0.0290 | 1.44 | 0.0068 | 2 |
| 1929 | P69905 | Hemagloblin subunit alpha HBA1 | 471 | 18 | 7 | 15305 | 8.05 | 16000 | -1.92 | 0.0005 | -1.15 | 0.1300 | -1.85 | 0.0290 | 1.44 | 0.0068 | 2 |
| 1929 | P00915 | Carbonic anhydrase 1 CA1 | 58 | 4 | 3 | 28909 | 8.05 | 16000 | -1.92 | 0.0005 | -1.15 | 0.1300 | -1.85 | 0.0290 | 1.44 | 0.0068 | 2 |
| 1932 | P68871 | Hemagloblin subunit beta HBB | 558 | 19 | 9 | 16102 | 9 | 15000 | -1.86 | 0.0019 | -1.20 | 0.0520 | -2.18 | 0.0250 | 1.27 | 0.0950 | 2 |
| 1932 | P69905 | Hemagloblin subunit alpha HBA1 | 204 | 7 | 4 | 15305 | 9 | 15000 | -1.86 | 0.0019 | -1.20 | 0.0520 | -2.18 | 0.0250 | 1.27 | 0.0950 | 2 |
| 1942 | P68871 | Hemagloblin subunit beta HBB | 849 | 26 | 10 | 16102 | 7.64 | 16000 | -1.62 | 0.0011 | -1.05 | 0.5600 | -1.99 | 0.0140 | 1.06 | 0.6100 | 2 |
| 1942 | P69905 | Hemagloblin subunit alpha HBA1 | 250 | 8 | 5 | 15305 | 7.64 | 16000 | -1.62 | 0.0011 | -1.05 | 0.5600 | -1.99 | 0.0140 | 1.06 | 0.6100 | 2 |
| 1947 | P68871 | Hemagloblin subunit beta HBB | 621 | 17 | 8 | 16102 | 8.21 | 16000 | -1.84 | 0.0011 | -1.08 | 0.2700 | -1.74 | 0.0120 | 1.09 | 0.5800 | 2 |
| 1947 | P69905 | Hemagloblin subunit alpha HBA1 | 445 | 17 | 7 | 15305 | 8.21 | 16000 | -1.84 | 0.0011 | -1.08 | 0.2700 | -1.74 | 0.0120 | 1.09 | 0.5800 | 2 |
| 1948 | P69905 | Hemagloblin subunit alpha HBA1 | 245 | 9 | 5 | 15305 | 8.8 | 16000 | -2.03 | 0.0150 | -1.12 | 0.3900 | -1.65 | 0.1900 | 1.44 | 0.0800 | 2 |
| 1968 | P68871 | Hemagloblin subunit beta HBB | 461 | 14 | 6 | 16102 | 7.54 | 16000 | -1.52 | 0.0025 | 1.10 | 0.3600 | -1.79 | 0.0570 | -1.10 | 0.5200 | 2 |
| 1968 | P69905 | Hemagloblin subunit alpha HBA1 | 140 | 4 | 3 | 15305 | 7.54 | 16000 | -1.52 | 0.0025 | 1.10 | 0.3600 | -1.79 | 0.0570 | -1.10 | 0.5200 | 2 |
| 2005 | P04792 | Heat shock protein beta-1 HSPB1 | 913 | 28 | 12 | 22826 | 6.88 | 34000 | 1.52 | 0.0036 | -1.06 | 0.7500 | -1.29 | 0.0700 | 1.92 | 0.0011 | 2 |
| 2167 | P08559 | Pyruvate dehydrogenase E1 component subunit alpha, somatic form, mitochondrial PDHA1 | 66 | 3 | 2 | 43952 | 4.99 | 15000 | -1.81 | 0.0500 | 1.21 | 0.1200 | 1.26 | 0.4200 | -1.46 | 0.0018 | 2 |
| 2167 | P06733 | Alpha-enolase ENO1 | 52 | 2 | 2 | 47481 | 4.99 | 15000 | -1.81 | 0.0500 | 1.21 | 0.1200 | 1.26 | 0.4200 | -1.46 | 0.0018 | 2 |
| 2175 | P04792 | Heat shock protein beta-1 HSPB1 | 813 | 29 | 12 | 22826 | 5.11 | 15000 | 1.67 | 0.0025 | -1.39 | 0.2300 | -1.02 | 0.9300 | 2.08 | 0.0100 | 2 |
| 2179 |  | No identification |  |  |  |  | 4 | 73000 | 1.55 | 0.2300 | 2.42 | 0.0380 | 1.22 | 0.6800 | 3.17 | 0.0001 | 2 |


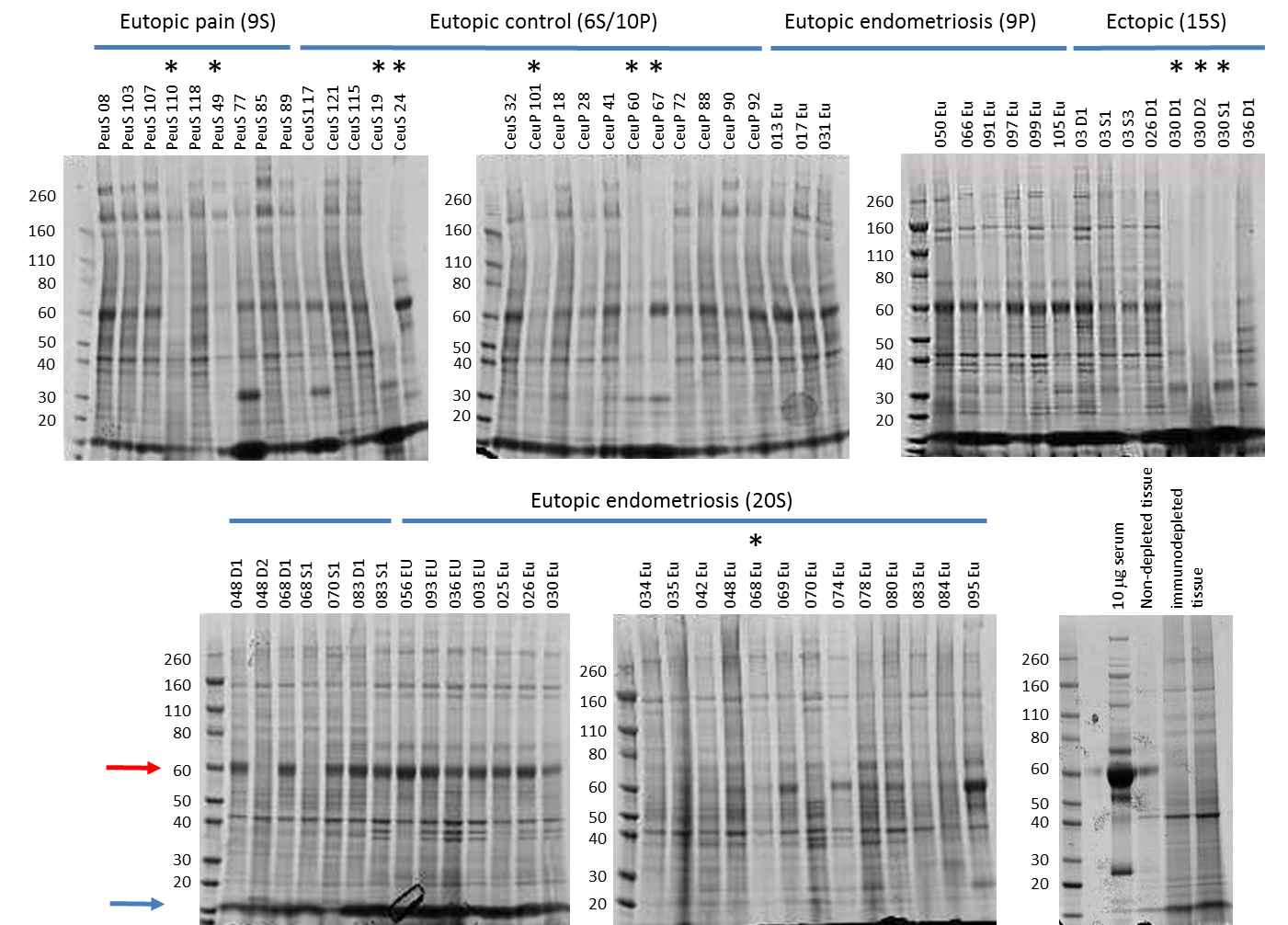


**Figure S1 Quality control of tissue lysates and immunodepletion test.** 10 μg of protein from each tissue lysate were run on 10% SDS-PAGE gels, stained with colloidal Coomassie Blue and bands compared with 10 μg of serum protein. It was clear that some of the samples were heavily contaminated with blood proteins, impairing the ability to visualise tissue-derived proteins, or had low protein staining overall. Samples denoted with an asterisk were subsequently excluded from further analysis. Examples of the highly abundant albumin and haemoglobin proteins are denoted by the red and blue arrow, respectively. The lower right-hand gel shows improvement in visualisation of ‘tissue’ proteins following immunodepletion of the 12 most abundant serum proteins using a commercial resin.


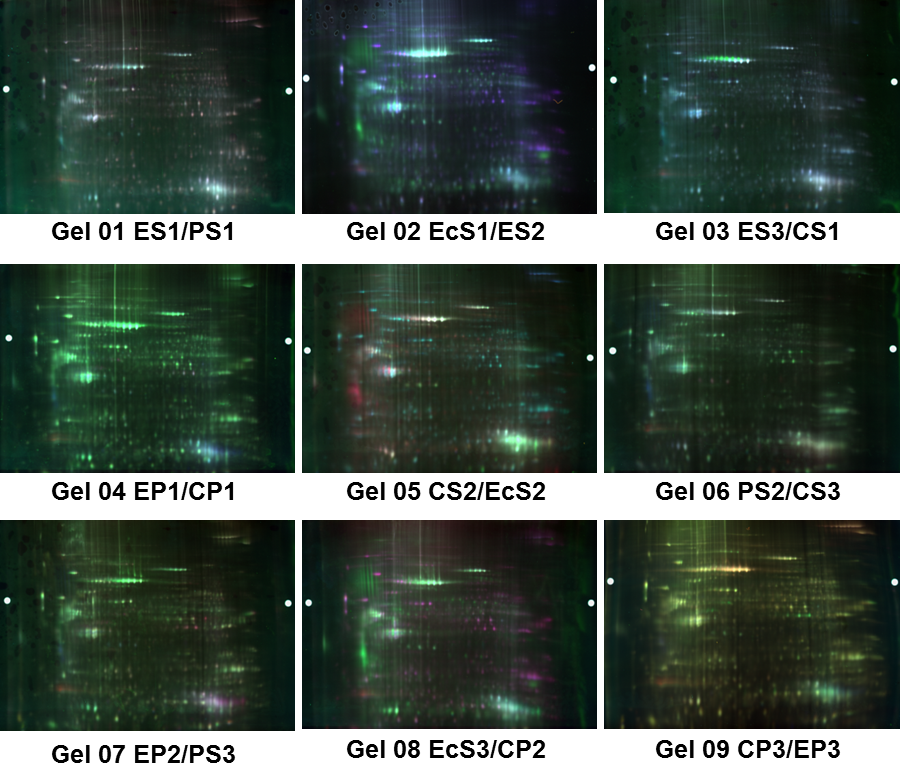


**Figure S2 Overlaid fluorescent 2D gel images from main 2D-DIGE profiling experiment.** Cy2, Cy3 and Cy5 fluorescent images are shown overlaid from a nine-gel experiment where pooled sample groups ES, EP, PS, CS, CP and EcS were run in triplicate. Differential expression was assessed using Decyder software**,** where spot matching across the nine gels was performed and standardised spot abundances calculated with reference to a Cy2-labelled standard pool (equal mix of all samples) run on all gels. Spot abundances were compared between clinical groups. Protein spots of interest were accepted if they displayed a >1.5-fold change in standardised abundance with *P*<0.05 (Student t test). These spots were picked from post-stained gels for identification by LC-MS/MS (see Table S2).
